# Haplotype analysis for Irish ancestry

**DOI:** 10.1080/20961790.2019.1639881

**Published:** 2019-09-12

**Authors:** Robert Whiting, Heather Miller Coyle

**Affiliations:** Forensic Science Department, Henry C. Lee College of Criminal Justice and Forensic Sciences, University of New Haven, West Haven, CT, USA

**Keywords:** Forensic sciences, forensic genetics, DNA, Y chromosome, haplotype analysis, DNA dragnet, familial search

## Abstract

Forensic haplotype analysis of the male Y chromosome is currently used to establish the number of male donors in sexual assaults, the number of male bleeders in blood pattern analysis, and for ancestry correlation to genetic founder populations in biogeographic studies. In forensic laboratory applications, its primary use is for DNA profile generation with trace amounts of male DNA in the presence of excess female DNA (e.g. spermatozoa identification, male component of fingernail scrapings). Our study supports the potential use of the Y chromosome in a “dragnet” approach (most haplotypes are unique) similar to that described by Kayser in 2017 for solving a cold case sex assault and homicide in The Netherlands. Our study also researched the potential for the identification of an ancestral Irish genetic “footprint” linked to surname O’Brien and identified multiple founder group origins in Ireland and England as well as three samples with the Dal Riata (a Gaelic overkingdom) ancestral haplotype. This study indicates correlation to ancestral Irish ancestry by haplotype but not conclusively to the O’Brien surname.

## Introduction

Genetic markers are used routinely for forensic applications and DNA databases [1–6]. Y-STRs are a specialty form of DNA testing that can be a useful tool for forensic applications. Y-STR markers are male specific, inherited as a genetic block, and often represent a common paternal lineage [7–10]. Over the years, Y chromosome-specific STRs have been used in criminal casework involving sexual assault with a multitude of different scenarios such as mixed stain samples, fingernail scrapings, sperm negative casework, cases of multiple male assailants and when evolutionary and genealogical background needs to be inferred [7–10]. Variations in DNA that are inherited together are known as haplotypes. Haplotypes are important because like surnames they are passed down generationally from father to son. Individuals who share a common surname might be expected to share more of their DNA if genetically related and, barring mutation, should be genetically identical by descent (IBD) [6,7]. For this project, the loci DYS19, DYS389I/II, DYS390, DYS391, DYS392, DYS393 and DYS385a/b (the European minimal haplotype), and loci DYS438 and DYS439 (recommended by the Scientific Working Group on DNA Analysis Methods (SWGDAM)) and locus DYS437, were used in the comparison of surnames [7,8]. A patrilineal surname is inherited in the same way as the non-recombining region of the Y chromosome, therefore, a correlation between the two should be recognized [7–14].

There are several Irish ancestry projects related to biogeographical ancestry. Three major ones are described here. The Ireland Y-DNA Project has 8 580 number break noted and offers both Y-chromosome (paternal) testing and mitochondrial DNA (maternal) testing to add to verification of historical information regarding ancestry. One hundred and eighty-one samples with surname O’Brien are included in this project (https://www.familytreedna.com/groups/ireland-heritage/about, accessed May 9, 2019). A second project researches the history of the Irish clans to identify founder populations and associated relatives many generations later. The goals of the Irish clan Y-DNA project are to connect genetic relatives and verify paternity and county of origin. Over 60 Irish clans are listed (http://www.clansofIreland.ie/baile/dna, accessed October 10, 2018). A third project has easily accessible haplotype data for identification of an O’Brien genetic footprint or common haplotype. The O’Brien surname project is available at website https://www.familytreedna.com/groups/obrien/about/background, accessed October 10, 2018). This website consists of sampled individuals correlated by geography, Y-chromosome haplotype and single nucleotide polymorphisms (SNPs).

## Materials and methods

Participants in our study were selected based on surname and ancestry and were asked to fill out a questionnaire (Appendix I) to gather background information (with informed consent). Buccal samples were collected and processed for male DNA haplotypes. Sample collection was performed according to University of New Haven guidelines and after Institutional Review Board (IRB) approval. Following human sampling, DNA from cotton swabs was extracted using the QIAamp^®^ DNA Mini kit (Qiagen; Germantown, MD, USA) following the buccal swab spin protocol [15]. DNA extractions were quantified using an ABI Quantifiler™ total Human DNA Quantification kit (according to the manufacturer’s recommendations; ThermoFisher Scientific, Waltham, MA, USA). Real-time PCR was performed on an Applied Biosystems 7500 Real-Time PCR system (ThermoFisher Scientific). All samples were amplified following the AmpFℓSTR^®^ Yfiler^®^ PCR Amplification kit (ThermoFisher Scientific) manufacturer’s protocol on an Applied Biosystems PCR GeneAmp 9700 thermocycler (ThermoFisher Scientific). After PCR amplification, sample fragment separation and detection was performed on an Applied Biosystems 3130xl Prism^®^ Genetic Analyzer (ThermoFisher Scientific). The results were then analyzed using GeneMarker software (SoftGenetics, LLC; State College, PA, USA) to score alleles at each genetic locus. The haplotypes were transferred to Excel software for data analysis. A pair-wise analysis was performed on all the samples within each population. The resulting data were assessed for a common genetic footprint between specific populations. When reporting new match criteria with Y-chromosome polymorphisms, the DNA Commission of the International Society of Forensic Genetics (ISFG) recommends three methods: counting method, likelihood ratio and Bayesian statistic [16]. Our study used the counting method or “frequentist approach” to compare haplotypes from different populations. The haplotype frequency is defined by a simple equation: X/N, where X = number of times observed; N = number of samples in the database.

## Results

Ten O’Brien’s of Irish descent were able to be successfully sampled and analyzed (Table 1). From the interview questionnaires, all the donors identified as being Caucasian having their paternal origins beginning in Ireland were not adopted and birthplace varied. From the results obtained, the most common shared allele in the haplotype was a 13 at locus DYS393 and a 14 at locus DYS19 (90% of the sampled population, respectively). Interestingly, the O’Brien’s county of origin that bordered or was in close geographic proximity with an adjacent county had a greater percentage of shared haplotypes than those O’Brien’s with counties of paternal origin that were not near in geography. This illustrates the importance of geography in surname analysis as farming communities often maintain family relationships for generations in a restricted area due to land assets. All the sampled Irish O’Brien’s had a unique haplotype except one pairing from County Clare. Within the general Irish population all the male subjects were of the Caucasian ethnicity and 66.66% of the population sampled was of pure Irish descent (Table 2). The remaining 33.34% of the sampled population was admixture between Irish and other European countries. Like the O’Brien population, all the analyzed samples had their paternal lineages originating in Ireland. The paternal lineages originated from counties Dublin, Clare, Cork, Limerick, Mayo, Galway and Kerry. The locations of birth were varied. The randomly sampled control population’s nationality, country of paternal origin and location of birth was much more diversified than the other two sampled populations (Table 3). In this control group, 83.33% of the population self-identified as Caucasian, 13.34% self-identified as Hispanic and 3.33% self-identified as Asian. The paternal origins for this population covered different regions of Western and Eastern Europe, the Mediterranean, Oceania, the Caribbean, the Middle East and Central America. Birthplace locations varied.

**Table 1.** O’Brien surname haplotypes (OB1–OB9; OB11).

Locus	Haplotypes									
	OB1	OB2	OB3	OB4	OB5^a^	OB6^a^	OB7	OB8	OB9^b^	OB11^b^
DYS456	16	15	17	16	16	16	16	17	17	15
DYS389I	12	12	14	14	13	13	14	14	13	13
DYS390	24	22	23	23	25	25	24	24	25	25
DYS389II	28	28	30	31	29	29	31	31	29	29
DYS458	18	15	16	16	17	17	18	17	16	18
DYS19	14	14	14	14	14	14	14	15	14	14
DYS385	11,14	13,14	12,14	11,15	11,13	11,13	11,14	11,15	11,14	11,14
DYS393	13	12	13	13	13	13	13	13	13	13
DYS391	11	10	11	11	10	10	11	10	11	11
DYS439	12	12	13	13	12	12	11	10	13	11
DYS635	23	21	23	23	23	23	23	21	23	23
DYS392	13	11	13	13	14	14	13	11	13	13
YGATAH	12	10	13	12	12	12	12	11	12	12
DYS437	15	16	14	15	15	15	15	15	15,16	15
DYS438	12	10	12	12	12	12	12	10	12	12
DYS448	19	20	19	19	18	18	18	20	18	19

a OB5 and OB6 are not known to be genetically related but both originate from County Clare, Ireland.

b OB9 and OB11 are consistent with the Dal Riata founder haplotype with the exception of the DYS389II locus.

**Table 2.** Irish haplotypes (I1–I9; I11).

Locus	Haplotypes									
	I1	I2	I3	I4	I5	I6	I7	I8	I9^a^	I11
DYS456	15	15	16	16	15	18	15	16	16	15
DYS389I	13	13	12	13	13	15	13	13	13	13
DYS390	24	23	24	24	24	24	24	23	25	24
DYS389II	29	29	29	29	30	33	29	30	29	29
DYS458	17	15	16	17	17	17	18	17	17	17
DYS19	15	14	14	14	14	15	14	15	14	14
DYS385	11,14	14,17	11,14	11,14	11,14	12,16	11,14	11,15	11,13	10,14
DYS393	13	12	13	13	14	13	13	13	13	13
DYS391	11	11	11	10	10	11	11	11	11	11
DYS439	11	12	12	13	12	11	11	12	12	11
DYS635	23	22	23	23	23	21	23	23	23	23
DYS392	13	11	13	13	13	12	13	13	14	13
YGATAH	12	12	12	11	11	11	13	11	12	12
DYS437	15	14	15	15	15	15	15	15	15	15
DYS438	12	9	12	12	12	10	12	12	12	12
DYS448	19	21	19	19	19	20	19	19	18	19

a I9 is consistent with the Dal Riata founder haplotype with the exception of the DYS389II locus.

**Table 3.** Caucasian haplotypes (C1–C9; C11–C18; C20; C23–C25; C28).

Locus	Haplotypes																					
	C1	C2	C3	C4	C5	C6^a^	C7^a^	C8	C9	C11	C12	C13	C14	C15	C16	C17^b^	C18^b^	C20	C23	C24	C25	C28
DYS456	14	15	14	15	16	16	16	16	15	15	16	17	17	16	15	15	15	15	17	17	15	15
DYS389I	12	13	14	14	14	13	13	13	13	13	13	13	14	12	12	14	14	13	14	14	13	13
DYS390	22	23	23	24	25	25	25	24	24	21	23	24	24	23	23	25	25	24	25	25	24	24
DYS389II	28	31	33	30	30	29	29	29	29	32	29	30	30	29	29	31	31	29	31	31	29	30
DYS458	15	17	17	17	17	16	17	19	17	17	18	15	16	17	18	16	16	17	17	17	17	17
DYS19	14	14	14	14	14	14	14	14	14	16	14	13	15	14	14	15	15	14	14	14	14	16
DYS385	12,14	13,20	13,16	11,14	11,14	11,14	11	10,14	11,15	16	11,14	16,18	11,14	16	13,16	11,14	11,14	11,14	11,13	11,13	11,15	14,15
DYS393	13	14	14	13	13	13	13	13	13	14	13	13	13	13	12	13	13	13	13	13	13	13
DYS391	10	10	10	11	10	11	11	10	10	10	10	10	10	10	10	11	11	11	11	11	10	11
DYS439	10	10	11	14	12	11	12	12	11	14	11	12	11	13	11	10	10	13	12	12	11	12
DYS635	24	24	22	23	23	23	23	24	24	21	23	24	23	21	24	23	23	23	23	23	23	23
DYS392	10	10	12	13	13	13	13	13	13	11	13	11	11	13	11	14	14	13	14	14	13	11
YGATAH	11	11	11	12	12	12	12	11	12	11	12	12	12	10	12	12	12	12	12	12	12	11
DYS437	16	16	14	15	15	15	15	14	15	14	14	14	14	14	15	14	14	14	15	15	15	15
DYS438	11	11	10	12	12	12	12	12	12	11	12	10	11	9	9	10	10	12	12	12	12	10
DYS448	19	19	20	19	18	19	19	18	19	20	19	20	19	18	20	19	19	19	18	19	19	20

a C6 and C7 are consistent with the Dal Riata founder haplotype with the exception of the DYS389II locus.

b C17 and C18 are known to be genetically related as a father-son pair and are expected to have the same paternal lineage.

After the pair-wise analysis of the randomly sampled O’Brien population was completed, trends were reviewed to determine if there were any similarities in males with varying locations of paternal origins and locations of origins of birth (Table 4). In the remainder of the sampled population there was an abundance of haplotype dissimilarity as one might anticipate with unrelated paternal lineages that lack a geographic connection. This control population indicates greater haplotype uniqueness and suggests that the Y-STR haplotype may be useful for screening individuals as a “dragnet” approach to identifying or eliminating persons of interest in a crime scene investigation (Table 5). Although Y-STR haplotypes have not been used to search national DNA databases for candidate matches due to population frequency issues, for crime scenes with a geographic restriction and required access to a victim, this may be a quick approach to narrowing the list of potential unrelated male individuals to fully investigate.

**Table 4.** Percent similarity (%) of a pair-wise comparison of O’Brien surname haplotypes.^a^

	OB1	OB2	OB3	OB4	OB5	OB6	OB7	OB8	OB9	OB11
OB1										
OB2	25.00									
OB3		6.25								
OB4			68.75							
OB5				43.75						
OB6					100.00					
OB7						50.00				
OB8							31.25			
OB9								12.50		
OB11									68.75	

a All O’Brien surnames identified as being Irish paternal descent; some individuals identified county of origin (OB1, Kerry; OB3, OB11, Limerick; OB5, OB6, Clare; OB8, Roscommon). Birthplace for OB5 and OB6 was West Haven, CT; all other samples varied. Paired samples OB5 and OB6 are the only pairing that had the same coincidental haplotype, county of paternal origin and birthplace.

**Table 5.** Haplotype profile probability estimates for O’Brien surname samples from US Y-STR Database (1 in *n* individuals).^a^

Ancestry	Haplotype frequencies									
	OB1	OB2	OB3	OB4	OB5	OB6	OB7	OB8	OB9	OB11
African American	2 083	2 083	2 083	2 083	2 083	2 083	2 083	2 083	2 083	1 316
Asian	1 335	1 335	1 335	1 335	1 335	1 335	1 335	1 335	1 335	1 335
Caucasian	1 613	2 488	2 488	2 488	1 613	1 613	2 488	2 488	2 488	1 613
Hispanic	758	1 592	1 592	1 592	1 592	1 592	1 592	1 592	1 592	1 592
Native American	758	1 190	1 190	1 190	1 190	1 190	1 190	1 190	1 190	1 190
Total	2 941	8 696	8 696	8 696	5 556	5 556	8 696	8 696	8 696	4 167

a Although mathematically profile frequency estimates indicate the Y-STR profile may repeat within a nationwide population for genetically unrelated individuals, purposeful random sampling in a restricted geographic location shows infrequent coincidental matching.

A potential relationship between surnames and Y-STR haplotype was demonstrated in a study conducted by Sykes and Irven [17]. In this study, the surname Sykes was randomly selected and typed for four microsatellites. Based on their results, researchers were able to conclude that there was a link between the Sykes surname population and the four typed microsatellites and haplotype expression. This common “genetic footprint” of 15-23-11-14 (corresponding to genetic loci DYS19, DYS390, DYS391 and DYS393) was not observed in either of the two control populations used in their study. The researchers concluded that there might be a single paternal ancestor from a founder population from which the Sykes surname originated. Our study was designed similarly to determine if the O’Brien surname could exhibit a similar unique haplotype distribution pattern. The Irish have been studied extensively for their haplotype origins and some ancestral groups such as the Dal Riata date back to approximately 500 AD. This founder population haplotype is 13-25-14-11-13-16 (DYS393-DYS390-DYS19-DYS391-DYS389I-DYS389II). A geographic map of common Irish haplotypes has been published indicating that within the confines of Ireland, a haplotype may be used as a predictor of county of origin. This likely is due to the island nature of Ireland and, although conquered repeatedly, groups of founder individuals remained to repopulate the regions. The O’Brien surname project is available at website https://www.familytreedna.com/groups/obrien/about/background. This is a large Irish population database that is being genotyped for common genetics based on a surname and is a larger dataset of independently collected and analyzed O’Brien samples. The O’Brien surname project has a common “genetic footprint” of 13-24-29-17-14-(11-14)-13-11-11-13-11-15-12-19 (DYS389I-DYS390-DYS389II-DYS458-DYS19-DYS385-DYS393-DYS391-DYS439-DYS392-YGATAH-DYS437-DYS438-DYS448). Our samples represent Irish that have emigrated out of Ireland to the US. Our 10 samples did not share this exact O’Brien surname “genetic footprint”, but remnants of the footprint remained within the haplotype. Percent sharing of the O’Brien surname alleles for our samples was: DYS389I (40%), DYS390 (30%), DYS389II (40%), DYS458 (30%), DYS19 (90%), DYS385 (80%), DYS393 (90%), DYS391 (60%), DYS439 (20%), DYS392 (60%), YGATAH (10%), DYS437 (70%), DYS438 (80%), DYS448 (40%). This allele frequency was then compared to 10 randomly sampled Caucasian with no O’Brien surname connection. Percent sharing of the general Caucasian sampling with the O’Brien surname alleles was: DYS389I (60%), DYS390 (30%), DYS389II (40%), DYS458 (70%), DYS19 (90%), DYS385 (50%), DYS393 (70%), DYS391 (30%), DYS439 (40%), DYS392 (60%), YGATAH (50%), DYS437 (50%), DYS438 (60%), DYS448 (50%). This information suggests that the most common alleles (14 at DYS19; 13 at DYS393; 13 at DYS392) are more fixed in the overall population while the residual O’ Brien surname “genetic footprint” may be only loosely correlated after emigration.

## Discussion

Our best interpretation is that the Y-chromosome and O’Brien surname is evolving over generations and once geographically unrestricted to Irish birthplace, only portions of the O’Brien surname “genetic footprint” remain. In fact, this is the strategy used for determining genetic relationships by paternal ancestry and is used routinely in evolutionary studies and family tree analyses. When comparing our O’Brien surname samples to the Oxford Genetic Atlas Project (OGAP) data in the Campbell study for R1b haplotypes, three samples carry the Irish ancestral signature of the Dal Riata, two samples bear the Northern Isles signature, two samples are consistent with Northern England and one sample is consistent with Central England [18]. The remaining samples do not bear an exact OGAP genetic signature. This dataset suggests that the Irish O’Brien that our study sampled originate from different founder populations. The haplotype frequencies for the O’Brien samples are presented in Table 5. If used as a DNA dragnet tool, one can see the frequency that one would expect to find a random match in the general unrelated population is low if one also considers the prior probability of an individual having access to the crime scene [19]. Haplotype analysis will not distinguish between genetic relatives with shared paternal lineages but all male relatives with access to the crime scene would be automatically added to the list of potential candidates to further investigate.

## Appendix I Questionnaire for Human Subjects Collection

1. If you agree to participate in this research project, would you be willing to supply a buccal swab?
2. What is your ethnicity or race?
  - Asian or Pacific Islander: all persons having origins in any of the peoples of the Far East, Southeast Asia, the Indian subcontinent, or the Pacific Islands.
  - Black (not of Hispanic origin): all persons having origins in any of the Black racial groups of Africa.
  - Hispanic: all persons of Mexican, Puerto Rican, Cuban, Central or South American, or other Spanish culture or origin, regardless of race.
  - American Indian or Alaskan Native: all persons having origins in any of the original peoples of North America, and who maintain cultural identification through tribal affiliation or community recognition.
  - White (not of Hispanic origin): all persons having origins in any of the original peoples of Europe, North Africa or the Middle East.
3. What nationality would you describe yourself as? If more than one, please list.
4. To your knowledge, do you know if your last name was altered in any way when your family emigrated to the United States?
5. Do you know in which century your family immigrated to the United States?
6. Do you know which country your father’s side of the family comes from?
7. If you know where your father’s family comes from, do you know which state/county/province they come from in that country?
8. To your knowledge, do you know if you are related to anyone on campus or in the community?
9. To your knowledge, do you know where you were born (please be as specific as you can)?
10. To your current understanding, do you know if you were adopted or if any males on your father’s side of the family were adopted?
11. Which surname or category applies to you (O’Brien, Irish, Other)?
